# The complete chloroplast genome sequence of wild Japanese pepper *Tubocapsicum anomalum* Makino (Solanaceae)

**DOI:** 10.1080/23802359.2021.1950063

**Published:** 2021-07-15

**Authors:** Rui-Hong Wang, Min-Min Chen, Yun-Zhe Wu, Rui-rui Wang, Xian-Fei Xiao, Zhe-Chen Qi, Xiao-Ling Yan

**Affiliations:** a Zhejiang Province Key Laboratory of Plant Secondary Metabolism and Regulation, College of Life Sciences and Medicine, Zhejiang Sci-Tech University, Hangzhou, China; b Shaoxing Academy of Biomedicine of Zhejiang Sci-Tech University, Hangzhou, China; c Shanghai Chenshan Plant Science Research Centre, Chinese Academy of Sciences, Shanghai Chenshan Botanical Garden, Shanghai, China

**Keywords:** *Tubocapsicum anomalum*, chloroplast genome, phylogeny

## Abstract

As an important medicinal herb, no complete organelle molecular data has been reported for *Tubocapsicum anomalum*. In this study, the first complete chloroplast genome of *Tubocapsicum anomalum* Makino was sequenced and assembled. The genome is 155,802 bp in length and contained 124 encoded genes in total, including 75 protein-coding genes, 10 ribosomal RNA genes, and 39 transfer RNA genes. The phylogenomic analysis showed that *Tubocapsicum anomalum* was closely related to *Withania somnifera* according the current sampling extent.

*Tubocapsicum anomalum* Makino 1908 (Solanaceae) is a medicinal herb with small yellow flowers and red juicy berries (Arcy et al. 2001). It is distributed in East Asia and Southeast Asia (Wang et al. 2018). Previous studies have indicated that *Tubocapsicum anomalum* may be developed as a novel class of chemopreventive drugs for the treatment of human cancers (Chang et al. 2007; Khan et al. 2012). Additionally, *Tubocapsicum anomalum* can also treat gonorrhea, dysentery and nephritis. As far as we know, there are no complete molecular data reports on organelles for *Tubocapsicum anomalum*. In this study, the first complete chloroplast genome of *Tubocapsicum anomalum* was reported. It will provide a better understanding on the evolution and genetics of Solanaceae.

The leaves of *T. anomalum* was collected from Chun’an, Zhejiang, China (GPS: E119°1′7.3″, N29°36′3.6″). The specimen and extracted DNA was deposited at the herbarium of Zhejiang Sci-Tech University (Zhejiang Province Key Laboratory of Plant Secondary Metabolism and Regulation, http://sky.zstu.edu.cn/, ZheChen Qi, zqi@zstu.edu.cn) under the voucher number ZSTU02769. The total genomic DNA was extracted from its silica dried leaves using DNA Plantzol Reagent (Invitrogen, Carlsbad, CA, USA) in accordance with the manufacturer’s instructions. The plastome sequences were generated using the Illumina HiSeq 2500 platform (Illumina Inc., San Diego, CA, USA). In total, *ca*. 14.6 million high-quality clean reads (150 bp PE read length) were generated with adaptors trimmed. Aligning, assembly, and annotation were conducted by GetOrganelle v1.7.0c, BLAST, GeSeq (Tillich et al. 2017) and GENEIOUS v11.0.5 (Biomatters Ltd, Auckland, New Zealand).

The full length of *T. anomalum* chloroplast sequence (GenBank Accession No. MW829600) is 155,802 bp, consisting of a large single-copy region (LSC with 86,260 bp), a small single-copy region (SSC with 18,400 bp), and two inverted repeat regions (IR with 25,571 bp). The GC content of *T. anomalum* chloroplast genome was 37.8%. A total of 124 genes were contained in the genome (75 protein-coding genes, 10 rRNA genes, and 39 tRNA genes). Seventeen genes had two copies, which were comprised of fine PCG genes (*ndhB, rps7, ycf2, rpl2, rpl23*), eight tRNA genes (*trnI-CAU, trnV-GAC, trnI-GAU, trnA-UGC, trnR-ACG, trnN-GUU, trnL-CAA, trnM-CAU*), and all four rRNA species (*rrn16, rrn23, rrn4.5, rrn5*). In the genome, eight protein-coding genes (*atpF, rpl2, ndhB, ndhA, rps16, rps12, rpoC1, petD*) had one intron, and *ycf3* gene contained two introns.

To confirm the phylogenetic position of *Tubocapsicum anomalum*, we obtained 14 published chloroplast genomes of Solanaceae and one accession of Convolvulaceae from NCBI. The sequence alignment was conducted using MAFFT v7.3 (Katoh and Standley 2013). The maximum likelihood (ML) phylogenetic analyses were constructed using IQTREE v1.6.7 (Nguyen et al. 2015), with the best selected TVM + F+R3 model and 5000 bootstrap replicates. The phylogenetic tree revealed that *T. anomalum* was closely related to *Withania somnifera* according the current sampling extent (Figure 1).

**Figure 1.**
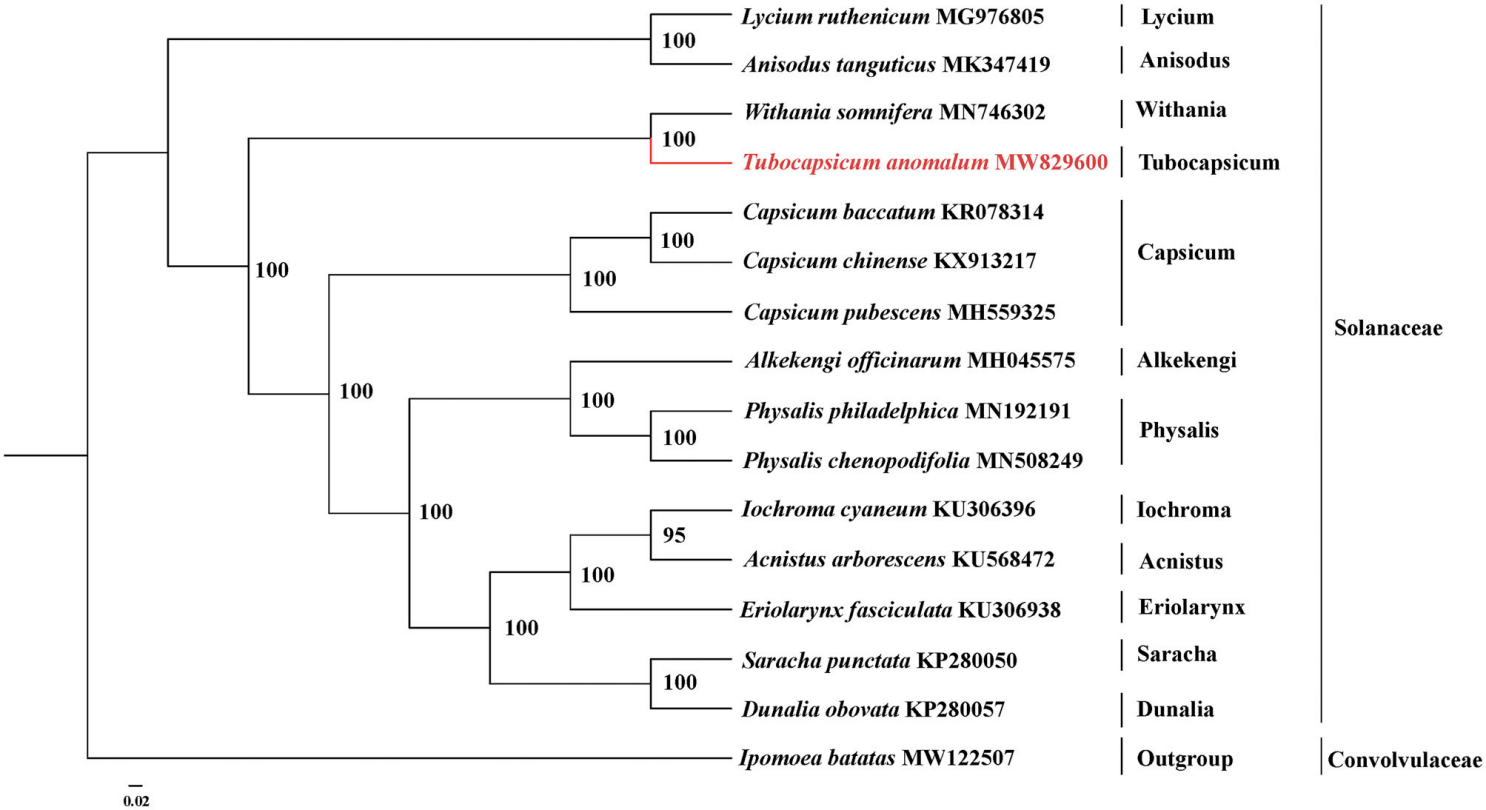
Phylogeny of Solanaceae based on complete chloroplast genomes (accession numbers were listed behind each taxon. Statistical support values were showed on nodes.

## Data Availability

The genome sequence data that support the findings of this study are openly available in GenBank of NCBI (https://www.ncbi.nlm.nih.gov) under the accession no. MW829600.The associated BioProject, SRA, and Bio-Sample numbers are PRJNA721932, SRR14237214, and SAMN18740964, respectively. The DNA matrix and phylogenetic tree that support the findings of this study are openly available in Github at https://github.com/andresqi/Tubocapsicum-anomalum-chloroplast-geome.
